# The role of CTHRC1 in promotion of cutaneous wound healing

**DOI:** 10.1038/s41392-022-01008-9

**Published:** 2022-06-15

**Authors:** Xianlan Duan, Xingyu Yuan, Bin Yao, Wei Song, Zhao Li, Enhejirigala, Yi Kong, Yuzhen Wang, Xiaobing Fu, Sha Huang

**Affiliations:** 1 School of Medicine, NanKai University, 94 Wei Jin Road, Tianjin, 300071 People’s Republic of China; 2 Research Center for Tissue Repair and Regeneration affiliated to the Medical Innovation Research Department, PLA General Hospital, 51 Fu Cheng Road, Beijing, 100048 People’s Republic of China; 3 Academy of Medical Engineering and Translational Medicine, Tianjin University, 92 Weijin Road, Tianjin, 300072 People’s Republic of China

**Keywords:** Trauma, Translational research

**Dear Editor**,

Wound healing after tissue injury is a well-orchestrated process involving various cell types, growth factors, and extracellular matrix (ECM) components. Collagen triple helix repeat containing-1 (CTHRC1), a secreted ECM protein, is transiently expressed in the repair process after arterial injury and myocardial infarction,^1^ regulating collagen matrix deposition and cell migration through TGF-*β* signaling pathway.^2^ However, the expression and function of CTHRC1 during cutaneous wound repair remain largely undefined.

We initially investigated the spatiotemporal expression of CTHRC1 during full-thickness cutaneous wound healing. By searching the GEO database, we found a transcriptome dataset (GSE23006), which analyzed the gene expression of mouse skin wounds from day 0 to day 10, spanned all stages of the wound healing process. Gene expression of *Cthrc1* was significantly increased from day 3, peaked at day 5, and fell back at day10 after injury (Fig. 1a). To verify this result, we examined the expression of CTHRC1 during wounding healing by immunofluorescence. CTHRC1 was weakly positive in the epidermis and hair follicles of normal skin. After wounding, CTHRC1 expressed in all dermis of the wound and appeared an intense increase at day 7 (Fig. 1b). We also verified these findings at the mRNA level by qPCR (Fig. 1c) and protein level by ELISA (Fig. 1d). Whereas our wound model was induced by an 8 mm punch biopsy, while the wounds of microarray were performed using a 1 mm punch biopsy. Therefore, when CTHRC1 was significantly upregulated in these two studies, wound healing was in the proliferative phases. This period involves the formation and maturation of granulation tissue, neovascularization, and re-epithelialization. Previous studies have revealed that CTHRC1 is significantly expressed on activated fibroblasts at 7 days after myocardial infarction,^3^ related to the pro-repair characteristics: increased cell proliferation and the deposition and synthesis of ECM molecules.^4^ The similar patterns of CTHRC1expression during skin repair suggests that the dynamic expression of CTHRC1 may also promote skin wound healing.

**Fig. 1:**
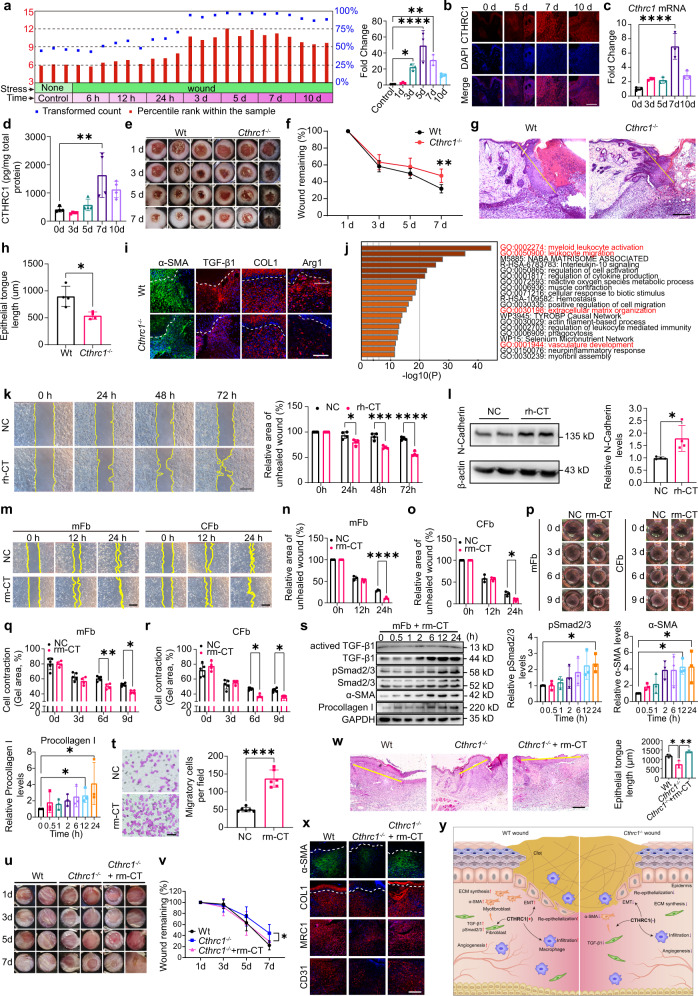
The role and mechanism of CTHRC1 in promoting cutaneous wound healing. **a** The relative expression values of *Cthrc1* mRNA during skin wound healing time course (0 d, 1 d, 3 d, 5 d, 7 d, and 10 d) were extracted from the existing RNA-seq gene expression dataset (GSE23006). *N* = 3 for each time-point. **b** Immunofluorescent staining for CTHRC1 from d 0 to d 10 post-wounding (CTHRC1, red; 4′,6-diamidino-2-phenylindole (DAPI), blue; scale bar, 100 μm). **c** *Cthrc1* mRNA expression was quantified in dermal tissues of the wound from 0 to 10 days post wounding using quantitative PCR. Values are given as mean ± SD. *N* = 3 for each time-point. **d** The CTHRC1 content of skin wound tissues was quantified by ELISA and normalized to the total protein concentration. *N* = 4 for each time-point. **e** Wounds were photographed at 1, 3, 5, and 7 days after wounding from 4 *Cthrc1*^−/−^ and 6 WT mice (Wt). **f** Quantification of wound area was from photographs. *Cthrc1*^−/−^ wounds are delayed in closure at day 7. Data are expressed as the percentage of the remaining area to the initial wound area. Values are given as mean ± SD. **g** Representative H&E stained sections of day 7 wounds from *Cthrc1*^−/−^ and WT mice. New epithelial tongue indicated by yellow lines (scale bar, 200 μm). **h** Quantification of the length of the new epithelial tongue (as demarcated by the yellow lines in **g**). Four sections were chosen for each group. Values are given as mean ± SD. **i** Immunofluorescence analysis of day 7 wounds from *Cthrc1*^−/−^ and WT mice. Sections were incubated with an antibody for α-SMA, TGF-β1, COL1, and Arg1 (α-SMA, green; TGF-β1, COL1, and Arg1, red; DAPI, blue; scale bar, 200 μm). **j** The top 20 GO term of the downregulated genes in the *Cthrc1*^−/−^ mice day 7 wounds compared to WT mice wounds by Metascape online tool. The color represents the enriched significance. **k** Left, representative images of the gap areas at 0, 24, 48, and 72 h after treating HaCaT cells with rh-CTHRC1 (100 ng/mL) in scratch wound healing assay (scale bar, 500 μm). Right, quantification of the scratch wound areas. Data are expressed as the percentage of the remaining area to the initial scratch area. Values are given as mean ± SD. **l** Left, western blot analysis of lysates was carried out to quantify N-Cadherin protein level of HaCaT cells treated with rh-CTHRC1. Right, quantification of N-Cadherin protein levels relative to β-actin. Data are presented as mean ± SD, *N* = 4. **m** Representative images of the gap areas at 0, 12, and 24 h after treating mFbs and *Cthrc1*^−/−^ fibroblasts (CFbs) with rm-CTHRC1 (100 ng/mL) in scratch wound healing assay (scale bar, 500 μm). **n**, **o** Quantification of the scratch wound areas. Data are expressed as the percentage of the remaining area to the initial scratch area. **p** Representative gel pictures at 0, 3, 6, and 9 d after the treatment of mFbs and CFbs with rm-CTHRC1 (100 ng/mL) in gel contraction assay. **q**, **r** Quantification of the gel areas. Data are expressed as the percentage of the remaining area to the initial gel area. **s** Left, western blot analysis of lysates was carried out to detect protein levels of mFbs treated with rm-CTHRC1. Right, quantification of α-SMA, and procollagen I protein levels relative to GAPDH, and pSmad2/3 protein levels relative to Smad2/3. Data are presented as mean ± SD, *N* = 3. **t** Representative images and quantification of migratory cells after treating RAW264.7 cells with rm-CTHRC1 (100 ng/mL) in Transwell migration assay (scale bar, 100 μm). The number of migratory cells was measured from five randomly selected fields. **u** Wounds were photographed at 1, 3, 5, and 7 days after wounding from 10 *Cthrc1*^−/−^, 10 rm-CTHRC1-treated *Cthrc1*^−/−^, and 8 WT mice. **v** Quantification of wound area was from photographs. *Cthrc1*^−/−^ wounds are delayed in closure at day 7, and rm-CTHRC1 recovered the wound healing rate. Data are expressed as the percentage of the closed area to the initial wound area. Values are given as mean ± SD. **w** Left, representative H&E stained sections of day 7 wounds from *Cthrc1*^−/−^, rm-CTHRC1-treated *Cthrc1*^−/−^, and WT mice (scale bar, 200 μm). Right, quantification of the length of the new epithelial tongue (indicated by the yellow lines). **x** Immunofluorescence analysis of day 7 wounds from *Cthrc1*^−/−^, rm-CTHRC1-treated *Cthrc1*^−/−^, and WT mice (scale bar, 200 μm). Sections were incubated with an antibody for α-SMA, COL1, MRC1, and CD31. **y** Schematic overview of the pivotal role and mechanism of CTHRC1 in promoting healing during the proliferation phase of cutaneous wound repair. *P < 0.05, **P < 0.01, ***P < 0.001, ****P < 0.0001

To uncover the role of CTHRC1 expression changes in skin wound healing, the closure rate of full-thickness skin wounds was compared between CTHRC1-deficient and wild-type mice. On day 7 post-injury, wound healing in *Cthrc1*^−/−^ mice was significantly delayed (Fig. 1e, f). Consistent with this result, the epithelial migration distance of *Cthrc1*^−/−^ mice wounds was significantly shortened (Fig. 1g, h). This alteration in wound closure corresponds with the onset and peak of CTHRC1 expression in *Cthrc1*^+/+^ mice (Fig. 1b). It indicates that CTHRC1 may have the capacity to stimulate keratinocytes to migrate into the wound area. Moreover, we found that the loss of CTHRC1 down-regulated the expression of genes associated with the repair, such as α-SMA, TGF-β1, COL1, and Arg1 (Fig. 1i). Therefore, altered wound-closure kinetics in *Cthrc1*^−/−^ mice might be due to delayed re-epithelialization by reduced keratinocyte migration and a reduction in α-SMA-expressing myofibroblasts within the granulation tissue, and thus resulting in reduced wound contraction. These findings suggest that the upregulated CTHRC1 in skin wounds can promote wound repair.

To reveal the function of CTHRC1 more systematically, we evaluated the effect of CTHRC1 deletion on wound healing at the transcription level. Because the peak of CTHRC1 expression is on day 7 post-injury, we performed RNA-seq to investigate the gene expression in *Cthrc1*^−/−^ mice post-injury-7-day wound dermis (Supplementary Fig. S1). GO enrichment analysis showed that the absence of CTHRC1 can attenuate the leukocyte activation, muscle contraction, ECM organization, and vasculature development of the wound (Fig. 1j). It indicated that the absence of CTHRC1 may affect immune regulation, wound contraction, deposition of extracellular matrix, and angiogenesis during the wound healing process.

To further uncover the mechanisms of CTHRC1 function, we explored the effects of CTHRC1 on various cell types involved in wound repair. Consistent with previous studies, CTHRC1 can promote HaCaT cells migration (Fig. 1k).^5^ While knockdown the expression of CTHRC1 by transfection with siRNA (si-CT) slowed down cell migration (Supplementary Fig. S2c, d). Both *Cthrc1*^+/+^ and *Cthrc1*^−/−^ mouse primary keratinocytes migrated faster after adding recombinant CTHRC1 (Supplementary Fig. S2f–h). But the *Cthrc1*^−/−^ keratinocytes are more sensitive in response to rm-CTHRC1. Moreover, we found that keratinocytes increased expression of the EMT marker N-cadherin after treatment with CTHRC1 (Fig. 1l and Supplementary Fig. S2i). Therefore, the role of CTHRC1 in promoting the migration of epidermal cells in vitro and in vivo may partly attribute to the promotion of EMT.

Fibroblasts are ubiquitous in the connective tissues of every organ system, where they deposit and remodel ECM. We found that CTHRC1 slightly promoted *Cthrc1*^+/+^ fibroblasts proliferation (Supplementary Fig. S3a), but significantly increased *Cthrc1*^−/−^ fibroblasts proliferation (Supplementary Fig. S3b, g). While knockdown of *Cthrc1* in mFbs by transfected with siRNA inhibited cell proliferation (Supplementary Fig. S3e, h). Both *Cthrc1*^+/+^ and *Cthrc1*^−/−^ fibroblasts accelerated migration (Fig. 1m–o, Supplementary Fig. S3k–n) and enhanced contraction after exogenous CTHRC1 addition (Fig. 1p–r). Correspondingly, mFbs transfected with si-CT led to a marked reduction in cell migration (Supplementary Fig. S3i, j, o, p). In addition, CTHRC1 upregulated the expression of TGF-β1, α-SMA, and procollagen I, and promoted phosphorylation of Smad2/3 in fibroblasts (Fig. 1s). Multiple studies have demonstrated that TGF-β1 can increase the expression of α-SMA through phosphorylation of Smad2/3 and plays a major role in myofibroblast differentiation, and CTHRC1 can activate TGF-β signaling via an elevation in Smad2/Smad3 phosphorylation.^2^ Therefore, our data indicated that in the process of skin injury repair, upregulated CTHRC1 might accelerate the migration of fibroblasts to the wound bed to form granulation tissue and promote their differentiation into myofibroblasts by activating the classic TGF-β pathway, thereby promoting wound contraction and ECM deposition. Besides, CTHRC1 also accelerated the migration of macrophages in vitro (Fig. 1t).

To further verify the biological function of CTHRC1 in vivo, we replenished the recombinant CTHRC1 protein in the wound of *Cthrc1*^−/−^ mice. The wound healing curve revealed that CTHRC1 accelerated wound healing after replenishment in *Cthrc1*^−/−^ mice wounds (Fig. 1u, v). This effect peaked at day 7 post-injury, consistent with the restored re-epithelialization rate (Fig. 1w) and increased myofibroblast population (Fig. 1x). The addition of CTHRC1 also increased collagen synthesis, the population of M2 macrophages, and neovascularization (Fig. 1x).

In summary, we revealed the dynamic expression of CTHRC1 during full-thickness skin wound healing. By knockout mouse model in vivo and cellular in vitro testing, we proved that CTHRC1 could accelerate wound healing by promoting epidermal migration, increasing M2 macrophage infiltration, promoting myofibroblast differentiation, regulating ECM deposition (Fig. 1y). Importantly, our data suggest that regulating CTHRC1 expression may provide a potential therapeutic regimen for some chronic wounds characterized by fibroblast aging, insufficient angiogenesis, and immune disorders.

## Supplementary information

supplementary material

## Data Availability

The RNA -seq data generated during this study have been deposited in the NCBI’s Gene Expression Omnibus (GSE200576). Other data that support the findings of this study are available from the corresponding author upon reasonable request.
