# Supplementary material for: The role of CTHRC1 in promotion of cutaneous wound healing
Source: Signal Transduct Target Ther. 2022 Jun 15;7:183. doi: 10.1038/s41392-022-01008-9 (PMC9197944; doi:10.1038/s41392-022-01008-9)
Supplement: Supplementary file 1 — supplementary material [file 41392_2022_1008_MOESM1_ESM.docx]

**Supplemental Material for**

**The Role of CTHRC1 in** **Promotion of Cutaneous Wound Healing**

Xianlan Duan^1,2,#^, Xingyu Yuan^1,2,#^, Bin Yao^2,3^, Wei Song^2^ , Zhao Li^2^, Enhejirigala^2^, Yi Kong^2^, Yuzhen Wang^2^, Xiaobing Fu^2,^*, Sha Huang^2,^*

1. School of Medicine, NanKai University, 94 Wei Jin Road, Tianjin 300071, PR China.

2. Research Center for Tissue Repair and Regeneration affiliated to the Medical Innovation Research Department, PLA General Hospital, 51 Fu Cheng Road, Beijing 100048, PR China

3. Academy of Medical Engineering and Translational Medicine, Tianjin University, 92 Weijin Road, Tianjin, 300072, PR China

#: These authors contributed equally to this work.

*: These authors are Correspondence

***Correspondence:**

Xiaobing Fu, Email: [fuxiaobing@vip.sina.com](mailto:fuxiaobing@vip.sina.com); Tel: + 010-66939114

Sha Huang, Email: [stellarahuang@sina.com](mailto:stellarahuang@sina.com); Tel: +010-66867384

**This file includes:**

Materials and Methods

Table. S1

Fig. S1-S3

**Materials and methods**

**Mice**

The procedures on animals were approved by the Institutional Animal Care and Use Committee of Chinese PLA General Hospital (Beijing, China). C57BL/6J mice 6–8 weeks of age were purchased from SiPeiFu (Beijing) Biotechnology Co., Ltd. *Cthrc1*^−/−^ mice (Riken BioResource Center, Acc. No: RBRC03519, link: <https://knowledge.brc.riken.jp/resource/animal/card?brc_no=RBRC03519&__lang__=en>) were generously provided by Prof. Z. Zhang (Ren Ji Hospital, Shanghai Jiao Tong University) and were conventionally bred in our animal laboratory. All mice were housed at 23 ± 3 °C with a 12/12-light/dark cycle and received lab chow and water ad libitum.

**Wounding and measurement methods**

Mice (both male and female) were 6-8 weeks of age at the time of wounding. Before wounding, mice were anesthetized with 4 % chloral hydrate, hair was removed with Veet (Reckitt Benckiser), and skin was cleaned with sterile water. Full-thickness skin wounds were induced on the mid-back by an 8-mm punch biopsy. The injuries were covered with Tegaderm (3 M, USA) and splinted with a neoprene ring (10 mm inner diagram rim). On the 0d and 3d after the wound was created, 30 μl of PBS or 600 ng/mL rmCTHRC1(AdipoGen, USA) was administered onto each wound. Wound healing was assessed by imaging every other day for 7 days. Wound closure was analyzed by comparing the relative wound size at a given time to 1d-post-wounding size using ImageJ software.

**Histology, immunofluorescence**

At various time points, skin and wound tissue were either snap-frozen in liquid nitrogen or fixed in 4% paraformaldehyde and embedded in paraffin according to standard procedures. Hematoxylin and eosin staining (HE) were performed to assess the re-epithelialization of the wounds. The length of the epithelial tongue, the epithelial migration distance, was defined as the unilateral distance between the wound border and the migrating front of keratinocytes. For immunofluorescence, slide samples were treated with 10% normal goat serum to block nonspecific epitopes for one hour. And samples were incubated with primary antibodies overnight at 4°C for anti-CTHRC1(1:200, ab85739, Abcam, USA), anti-TGF-β1(1:300, ab215715, Abcam, USA), anti-α-SMA(1:200, ab7817, Abcam, USA), anti-CD31(1:200, ab24590, Abcam, USA), anti-MRC1(1:400, ab64693, Abcam, USA), anti-COL1 (1:500, ab21286, Abcam, USA) and anti-Arg1(1:200, ab96183, Abcam, USA). Fluorophore-conjugated secondary antibodies were incubated for 2 h at RT in the dark. Nuclei were labeled with DAPI (SouthernBiotech, USA). Immunofluorescence stainings were analyzed using a confocal microscope (TCS SP8 STED, Leica, Germany).

**Cell Culture and treatment**

Primary dermal fibroblasts and keratinocytes were isolated from the skin of one-day-old mice as described^1^. Fibroblasts and HaCaT cells were cultured in Dulbecco's modified essential medium (DMEM) supplemented with 10% fetal bovine serum (Gibco, USA), and keratinocytes maintained in Keratinocyte Medium (Sciencell, USA). Cells were cultured at 37 °C in a humidified atmosphere of 5% CO2. To overexpress *Cthrc1*, the cells were treated with 100 ng/mL recombinant CTHRC1 (rm-CTHRC1: AG-40B-0154; rh-CTHRC1: AG-40B-0157; AdipoGen, USA). To knockdown *CTHRC1*/*Cthrc1*, cells were transfected with CTHRC1-small interfering RNA (si-CT, 50 nmol/L, Tsingke, China) and negative control (siRNA NC, Tsingke) by jetPRIME transfection reagent (Polyplus, USA).

**Gel contraction assay**

Cells (2×10^6^ cells/ml) were embedded in 150 μL gel containing rat tail collagen type 1 (a final collagen concentration: 2.5 mg/mL, Solarbio, China) and Matrigel (a final protein concentration: 2 mg/mL, Corning, USA), and were immediately added to the wells of a 48- well plate. The gels were allowed to polymerize at 37 °C for 30 min, then gently detached with a sterilized spatula and fresh growth media added. Images were captured at 0 d and every other day. The area of the gel was measured using ImageJ software.

**CCK-8 assay**

Cells (1 × 10^4^ in 100 μL) were seeded into a 96-well plate to measure proliferation with a cell counting kit 8 (CCK-8; Dojindo, Japan). Cell viability was assessed at 48h after treatment. And the cell proliferation was measured at 0, 12, 24, 48, and 72 h. The absorbance at a wavelength of 450 nm (OD450) was tested on a SPARK 10M reader (TECAN).

**EdU Cell Proliferation Assay**

The proliferation of cells was assessed by an EdU apollo 567 *in vitro* kit (Solarbio, China) according to manufacture instructions. The nuclei were stained with Hoechst 33342. EdU incorporation was evaluated by fluorescence microscopy (Leica DMI4000B). Cells double-labeled with Hoechst and EdU in the nuclei were considered as dividing cells.

**Cell migration assay**

The migration ability of mFbs was evaluated utilizing wound scratch assay and transwell assay. For wound scratch assay, mFbs were seeded in 6-well plates at 5 × 10^5^/well. When 90 % confluence was reached, cells were scratched perpendicularly using a pipette tip and washed with PBS 3 times. And the medium was changed to DMEM supplemented with 1% FBS to reduce proliferation. The closure of scratches was documented by optical microscopy every 12 h. For transwell assay, 2 × 10^4^ mFbs in serum-free DMEM were added on the upper side of the transwell chamber (3422, Corning, USA), while the complete medium was added to the lower chamber. After incubation for 24 h at 37°C, the chamber was fixed with 4% PFA for 30 min and stained with 0.1% crystal violet (Solarbio), followed by swabbed the non-invaded cells on the upper surface of the polycarbonate membrane. Then, 5 fields of view were randomly selected for observation and cell counting under optical microscopy.

**RNA isolation, reverse transcription, and quantitative real-time PCR**

After dissolution, the sample was fully immersed in Trizol reagent (Ambion, USA) for total RNA extraction**.** Total RNA (1 μg) was reverse transcribed into cDNA using a PrimeScript reverse reaction kit with gDNA Eraser, according to the manufacturer's protocols (Takara). Quantitative real-time PCR was performed using TB Green Premix Ex Taq II (Takara, China), on an ABI QuantStudio 5 system (Applied Biosystems, USA) with the following program: 95°C for 5 min, 40 cycles of 95°C for 10 s, 60°C for 34 s, and 72°C for 5 s, and 72°C for 10 min. *GAPDH*/*Gapdh* was used as the reference gene for normalization. The gene expression levels were quantified as the comparative cycle threshold (CT) method relative to *GAPDH*/*Gapdh* using the 2-ΔΔCT method. The primers of genes are shown in Table S1.

**Western blotting**

Cells were lysed with SDS lysis buffer containing protease inhibitors and phosphatase inhibitors (Roche). The primary antibodies included CTHRC1 (1:600, ab85739, Abcam, USA), TGF-β1 (1:1,000, ab215715, Abcam, USA), α-SMA (1:1,000, ab7817, Abcam, USA), Procollagen I (1:1,000, ab260043, Abcam, USA) , N-Cadherin (1:1,000, ab18203, Abcam, USA), Smad2/3 (1:1,000, ab202445, Abcam, USA), pSmad2/3 (1:1,000, 8828, CST, USA). The membranes were reprobed with anti-β-actin (1:5000, 66009-1-Ig, Proteintech, USA) or anti-GAPDH (1:5000, 60004-1-Ig, Proteintech, USA) as the loading control. An ECL detection system (Applygen, China) was used to display protein expression with a UVITEC Alliance MINI HD9 system (UVITEC, Britain), and protein expression was quantified using Image J software.

**Enzyme-linked immunosorbent assay**

The amount of CTHRC1 in the skin and wound tissues was quantified by the CTHRC1 ELISA Kit (JiYinMei, JYM1045Mo, China) and normalized to the total protein concentration measured by BCA assay (PC0020, Solarbio) according to the manufacturer's guidelines.

**Transcriptome expression and Gene Ontology enrichment analysis**

Freshly harvested skin or wound tissue was weighed and cut into 2-mm pieces followed by digested at 37°C for 30 min with 3 mg/mL dispase Ⅱ (Sigma-Aldrich, USA) to remove the epidermal layer. Total RNA was extracted from the wound tissue using Animal Tissue RNA Purification Kit (Norgen, Canada) according to manual instruction. RNA sequencing was performed use Illumina noveseq by LC Sciences. Genes with mean log intensity >2.0, fold change >2.0, and false rate < 0.05 were collected and considered significant. Gene Ontology (GO) analysis of differentially expressed genes (DEGs) was performed by Metascape. (<https://metascape.org/gp/index.html#/main/step1>).

**Statistical analysis**

The statistical significance between groups was calculated using the Student's t-test or one-way analysis of variance (ANOVA) when normality and equal variance were assumed. The statistical analyses were performed using SPSS 24. A P-value < 0.05 was considered statistically significant.

**Reference**

1 Zhang, Y. *et al.* Using bioprinting and spheroid culture to create a skin model with sweat glands and hair follicles. *Burns Trauma* **9**, tkab013, doi:10.1093/burnst/tkab013 (2021).

**Table S1**

| Primers | Sequences (5' to 3') | |
| --- | --- | --- |
| Cthrc1 | Forward | GCTGTCAGCGCTGGTATTTT |
|  | Reverse | AGCACCAATCCCTTCACAGA |
| Gapdh | Forward | AACGACCCCTTCATTGACCT |
|  | Reverse | ATGTTAGTGGGGTCTCGCTC |
| CTHRC1 | Forward | GGAATGTGCTTACAAGGGCC |
|  | Reverse | GTCCAGGACTCCTCAAAGCT |
| GAPDH | Forward | CTGACTTCAACAGCGACACC |
|  | Reverse | GTGGTCCAGGGGTCTTACTC |

**Fig.S1**

**
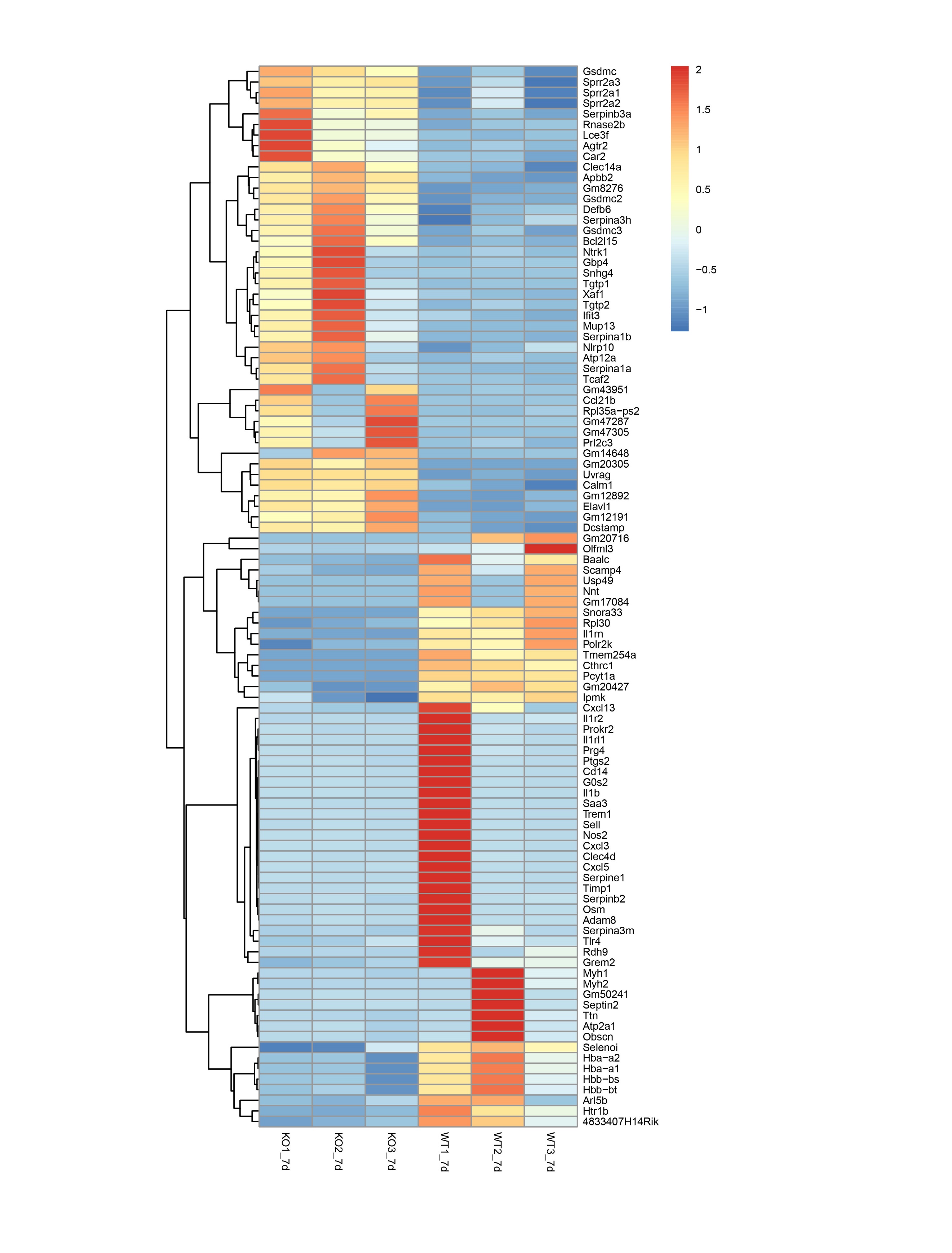
**

**Fig.S1** Clustered heatmap of the top 100 differentially expressed genes (DEGs) among dermis tissue of wound from *Cthrc1*^-/-^ and WT mice at day 7 post-injury.

**Fig.S2**

**
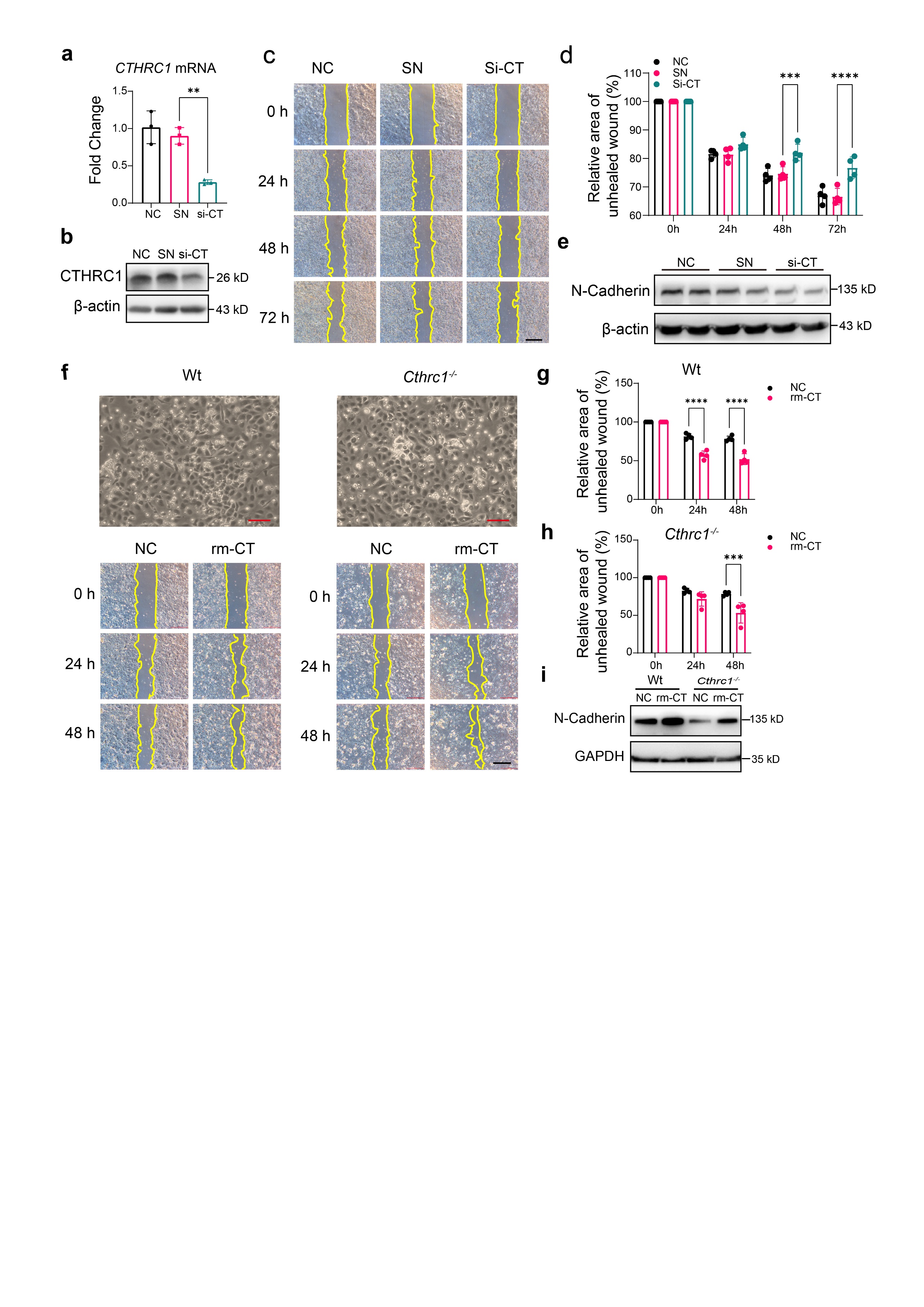
**

**Fig.S2 CTHRC1 promoted keratinocytes mobility *in vitro*. (a)** qPCR analysis of *CTHRC1* knockdown efficiency in HaCaT cells after 48 h transfection of control siRNA (SN) and *CTHRC1* siRNA (si-CT). **(b)** Western blot analysis of *CTHRC1* knockdown efficiency in HaCaT cells after 48 h transfection of SN and si-CT. Equal loading was confirmed by blotting for β-actin. **(c)** Representative images of the gap areas after the treatment of HaCaT cells with si-CT. **(d)** Quantification of the scratch wound area. Data are expressed as the percentage of the remaining area to the initial scratch area. Values are given as mean ± SD. **(e)** Western blot analysis of lysates was carried out to quantify N-Cadherin protein level of HaCaT cells treated with si-CT. Equal loading was confirmed by blotting for β-actin. **(f)** Representative images of the gap areas at 0, 24, 48, and 72 h after treating *Cthrc1*^+/+^ and *Cthrc1*^-/-^ mouse primary keratinocytes with rm-CTHRC1 (100 ng/mL) in scratch wound healing assay (scale bar, 500 μm). **(g, h)** Quantification of the scratch wound areas. Data are expressed as the percentage of the remaining area to the initial scratch area. Values are given as mean ± SD. **(i)** Western blot analysis of lysates was carried out to quantify N-Cadherin protein level of keratinocytes treated with rm-CTHRC1. Equal loading was confirmed by blotting for GAPDH. ****P* < 0.001, *****P* < 0.0001.

**Fig.S3**


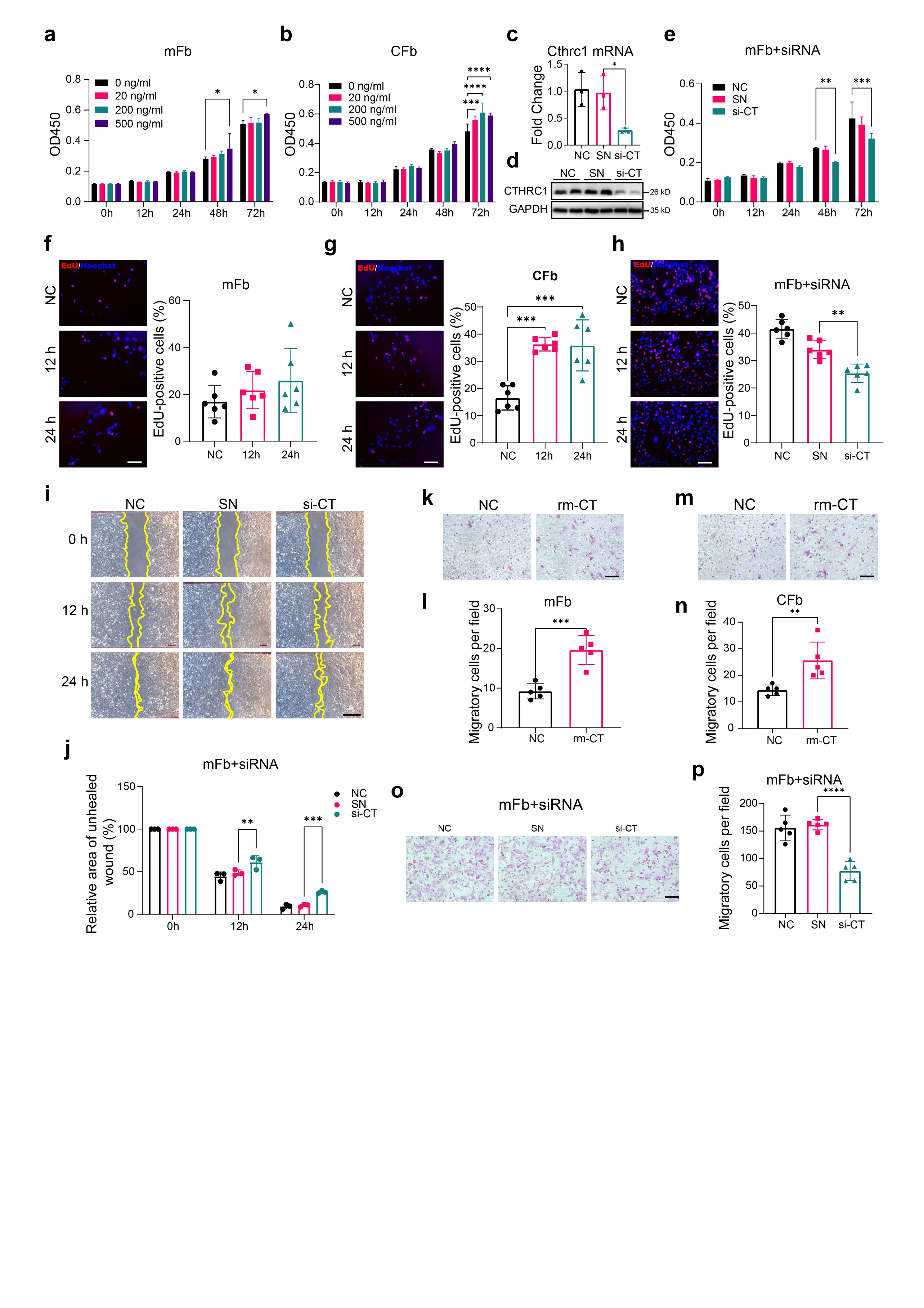


**Fig.S3 CTHRC1 influences fibroblast behavior. (a)** The effects of various concentrations of rm-CTHRC1 treatment on the viability of mouse fibroblasts (mFbs) for 12, 24, 48, and 72 hours were determined by CCK-8 assay. **(b)** The effects of CTHRC1 in the proliferation of *Cthrc1*^-/-^ fibroblasts (CFbs). **(c)** qPCR analysis of *Cthrc1* knockdown efficiency in mFbs after 48 h transfection of control siRNA (SN) and *Cthrc1* siRNA (si-CT). **(d)** Western blot analysis of *Cthrc1* knockdown efficiency in mFbs after 48 h transfection of SN and si-CT. Equal loading was confirmed by blotting for GAPDH. **(e)** The effects of *Cthrc1* knockdown on the viability of mFbs were measured by CCK-8 assay. **(f)** Representative images and the rates of mFbs proliferation after treatment with rm-CTHRC1 (100 ng/mL) for 12 and 24 h were detected by EdU incorporation assay (scale bar, 100 μm). The percentage of EdU-positive cells was measured from six randomly selected fields. **(g)** The percentages of CFbs proliferation after treatment with rm-CTHRC1 (100 ng/mL). **(h)** The rates of mFbs proliferation after *Cthrc1* knockdown were measured by EdU incorporation assay. **(i)** Representative images of the gap areas at 0, 12, and 24 h after treating mFbs with si-CT in scratch wound healing assay (scale bar, 500 μm). **(j)** Quantification of the scratch wound areas. Data are expressed as the percentage of the remaining area to the initial scratch area. **(k, m)** Representative images of migratory cells after treating mFbs and CFbs with rm-CTHRC1 (100 ng/mL) in Transwell migration assay (scale bars, 100 μm). **(l, n)** The number of migratory cells was measured from five randomly selected fields. **(o, p)** Migratory cells of mFbs were determined by Transwell assay after *Cthrc1* knockdown. **P* < 0.05, ***P* < 0.01, ****P* < 0.001, *****P* < 0.0001.
